# Thresholds for Phosphatidylserine Externalization in Chinese Hamster Ovarian Cells following Exposure to Nanosecond Pulsed Electrical Fields (nsPEF)

**DOI:** 10.1371/journal.pone.0063122

**Published:** 2013-04-29

**Authors:** Rebecca L. Vincelette, Caleb C. Roth, Maureen P. McConnell, Jason A. Payne, Hope T. Beier, Bennett L. Ibey

**Affiliations:** 1 National Research Council, Fort Sam Houston, Texas, United States of America; 2 Department of Radiological Sciences, University of Texas Health Science Center at San Antonio, San Antonio, Texas, United States of America; 3 Air Force Research Laboratory, Radio Frequency Bioeffects Branch, Fort Sam Houston, Texas, United States of America; Emory University School of Medicine, United States of America

## Abstract

High-amplitude, MV/m, nanosecond pulsed electric fields (nsPEF) have been hypothesized to cause nanoporation of the plasma membrane. Phosphatidylserine (PS) externalization has been observed on the outer leaflet of the membrane shortly after nsPEF exposure, suggesting local structural changes in the membrane. In this study, we utilized fluorescently-tagged Annexin V to observe the externalization of PS on the plasma membrane of isolated Chinese Hamster Ovary (CHO) cells following exposure to nsPEF. A series of experiments were performed to determine the dosimetric trends of PS expression caused by nsPEF as a function of pulse duration, τ, delivered field strength, E_D,_ and pulse number, n. To accurately estimate dose thresholds for cellular response, data were reduced to a set of binary responses and ED50s were estimated using Probit analysis. Probit analysis results revealed that PS externalization followed the non-linear trend of (τ*E_D_^2^)^−1^ for high amplitudes, but failed to predict low amplitude responses. A second set of experiments was performed to determine the nsPEF parameters necessary to cause observable calcium uptake, using cells preloaded with calcium green (CaGr), and membrane permeability, using FM1-43 dye. Calcium influx and FM1-43 uptake were found to always be observed at lower nsPEF exposure parameters compared to PS externalization. These findings suggest that multiple, higher amplitude and longer pulse exposures may generate pores of larger diameter enabling lateral diffusion of PS; whereas, smaller pores induced by fewer, lower amplitude and short pulse width exposures may only allow extracellular calcium and FM1-43 uptake.

## Introduction

The disruption of plasma membranes by *in situ* micro- and millisecond electrical pulses has been observed for several decades, but the exact mechanism(s) responsible for this disruption remains unknown [1], [2], [3]. A well-supported hypothesis suggests that electrical pulses cause dielectric breakdown of the plasma membrane, resulting in the formation of transient pores [3], [4], [5], [6], [7], [8], [9], [10], [11], [12], [13]. This hypothesis is bolstered by molecular dynamic models of pure lipid bilayer showing the formation of water pores within nanoseconds after the application of an electric field [14], [15]. A recent paper has modeled this phenomenon on the cellular scale and concluded that short duration (nanosecond), high intensity pulses likely create a large population of small pores, whereas longer duration, low amplitude pulses create large pores [16]. Interestingly, the lifetime of these nanopores has been empirically measured to be on the order of minutes following a single 60 ns exposure [3], [11], [17], [18], [19]. Such an extended lifetime may permit secondary chemical pathways to activate, resulting in changes in membrane confirmation not directly related to the pulse.

Electrical pulses have been demonstrated to externalize PS from the inner to the external leaflet of the cellular plasma membrane [1], [20], [21]. It is energetically unfavorable for a PS head group to flip, or translocate, to the external leaflet of the membrane [22], thus PS will exist predominantly in the inner leaflet of a healthy cell unless enzymatic pathways dictate otherwise. Two enzymes, scramblase (Ca^2+^ dependent) and translocase (Ca^2+^ inhibited, ATP-dependent), maintain the asymmetrical distribution of PS in the bilayer membrane [23]. However, upon application of mechanical or electrical stresses that porate the membrane, lateral diffusion of PS is energetically achievable. Using molecular dynamics (MD) models, Levine et al. determined that lateral diffusion could move PS from the inner to outer portion of the membrane in what they defined as the maturation phase, when the hydrophilic pore has been formed [24]. However, due to influx of extracellular calcium through nanopores, and hypothesized release of intracellular calcium stores, it is possible that these enzymes are being activated.

To address the possibility of enzymatic activation by an influx of calcium through nanopores, Vernier et al. used FM1-43, a membrane-reactive dye commonly used to monitor exocytosis in neurons [25] that is not inherently calcium dependent. Since extracellular calcium is required for Annexin V binding to PS, by using FM1-43 it could be determined if disruption of the membrane persisted even without the extracellular calcium. Upon single cell exposure to nsPEF, the plasma membrane facing the poles showed increased fluorescent intensity. From this data, they hypothesized that externalization may be due to the lateral diffusion of PS through semi-stable nanopores in the plasma membrane. Vernier *et al.* [21] also presented PS externalization field-dependent data in which variable field strengths and pulse repetition rates were used to deliver 50 pulses of nsPEF to Jurkat cells. These data demonstrated that PS externalization is dependent on field strength and pulse repetition rate. Vernier *et al.* [21] hypothesized that multiple nsPEF may elicit a non-linear response in the membrane if the cellular disturbance lasts longer than the pulse repetition rate.

The mechanism behind acute PS externalization following nsPEF exposure remains unclear. However, tracking of PS externalization remains one of the key observations pinpointing a direct change in the molecular confirmation of the plasma membrane. Therefore, we sought to examine dosimetric trends in cells exposed to nsPEF for various field strengths, pulse durations and number of pulses delivered at a constant repetition rate to elucidate the “dose response” for PS externalization. We believe that the expression of PS on the exterior of the membrane occurs as a function of pulse settings, (pulse duration, τ, and electric field strength, E_D_) therefore, the number and size of induced membrane pores. Dosimetric trends of cells exposed to electrical pulses have been reported [21], [26], [27], [28], [29], but this is the first account of a quantifiable non-linear trend in PS externalization from an nsPEF in live-cells. Additionally, we have shown that rapid increases in intracellular calcium occur at doses far below that required for PS externalization. By simply comparing the dose requirement for each observation, we can conclude that nsEP-induced calcium release is unlikely related to PS externalization. Additionally, by comparing the thresholds across multiple pulse parameters for externalization of PS with FM1-43 dye uptake, we show that pore diameter may ultimately determine whether PS is able to externalize after electric pulse exposure. This result suggests that energetic barriers present in small diameter pores may inhibit lateral diffusion of PS.

## Materials and Methods

### Cell Culture

Chinese hamster ovary (CHO-K1) cells (ATCC# CCL-61, American Type Culture Collection, Manassas VA) were grown according to the supplier’s protocol. The cells were cultured in F-12K medium (ATCC, Cat# 30-2004) supplemented with 10% Fetal Bovine Serum (FBS) (ATCC, Cat# 30-2020) and with 1% penicillin/streptomycin (ATCC, Cat# 30-2300) and incubated in a 5% CO_2_ maintaining, 95%humidity environment at 37°C. Twenty-four hours before exposure, the cells were treated with trypsin, pelleted, counted by a Beckman Coulter Z1 particle counter (Brea, CA) and re-suspended to 500 cells/µL. Approximately 6.5×10^3^ cells were plated on 35-mm glass bottom dishes coated with Poly-d-lysine (MatTek Corporation, Ashland, MA, Cat# P35GC-0-10-C) and allowed to incubate in complete growth medium overnight at 37°C/5%–CO_2_/95% - humidity.

### FITC-Annexin-V, FM1-43, and Calcium Green AM-1 Labeling

To prepare for live cell imaging of PS externalization, cells plated the previous day were washed with 2 mL of warmed exposure buffer comprised of 2 mM MgCl_2_, 5 mM KCL, 10 mM HEPES, 10 mM Glucose, 2 mM CaCl_2_ and 135 mM NaCl. The exposure buffer had an osmolarity ranging from 290-310-mOsm and pH 7.4. We added 200 µL of FITC-Annexin-V (BD Pharmingen, San Jose, CA, Cat# 556419) and 4 µL (1 mg/mL) of propidium iodide (PI) (BD Pharmingen, Cat# 51-66211E) to 1.8 mL of warmed buffer (same as above), then filtered through a 0.22 µm syringe filter before being placed on the cells.

PI was used to indicate the overall health of the cell prior to exposure and only cells initially free of PI were selected. The samples were effectively “doped” with 10× the manufacturer’s suggested 10 µL prepared fluorescein isothiocyanate-labeled Annexin V (FITC-AV) per 1 mL buffer to ensure abundant availability of this fluorophore. The cells were covered and kept at room temperature in the dark for a minimum of 15-min before nsPEF exposure.

To detect uptake of FM1-43 dye into CHO-K1 cells, 10 µL of FM1-43 dye (Invitrogen, Carlsbad, CA) at 1 mg/mL in water, was added to 2 mL of exposure buffer and the cells were incubated for 15 minutes at room temperature.

To detect the influx of extracellular calcium, similarly plated CHO-K1 cells were washed twice with D-PBS (without calcium or magnesium). A fresh 4 µL aliquot of Stock Calcium Green 1 AM Ester (CaGr) of 50 µg in 13 µL DMSO (Invitrogen, Carlsbad, CA, Cat # C-3011 MP) was thawed in preparation for the staining. We added 4 µL of the prepared CaGr stock to 2 mL of outside solution covering the plated cells. The cells were covered and slowly rocked on an orbital shaker at room temperature for 60-min in the dark. Approximately 15 min before nsPEF exposure, 4 µL of PI stock was added to the plate, gently rocked for 15–30 sec then transported to the confocal microscope for experiments.

### Pulser, Electrodes and Dosimetry

**Figure 1:**
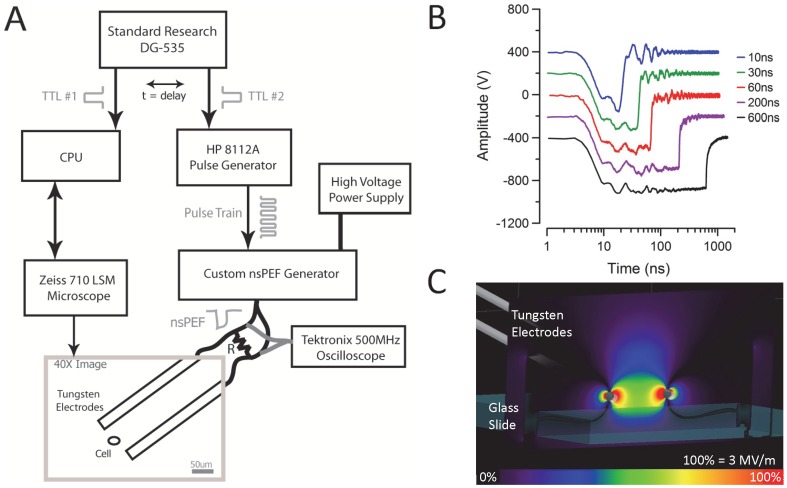
Application of nsPEFs to single cells. (A) The nsPEF exposure and imaging experimental set-up. An EC Plan-Neofluar 40×/1.3 DIC-oil Zeiss objective was used with the Zeiss LSM 710 microscope to image CHO-K1 cells, plated in a MatTek imaging plate, centered between the tungsten electrodes. The load resistor, R = 50 W. (B) Traces of the nsEP delivered by the system are shown as captured by a 500 MHz Tektronix oscilloscope connected across the load resistor. (C) A transverse section of the FDTD model for the tungsten electrode exposure system at 50 µm above the glass surface predicting an electric field of 2.3 MV/m at the cell for a 500V pulse amplitude.

Fig. 1A depicts our pulsing system. A Stanford DG535 digital delay generator generated two transistor-transistor logic (TTL) pulses with a preset time-delay. TTL #1 triggered the beginning of data acquisition by a Zeiss LSM 710 confocal microscope. The microscope captured a 512×512 pixel image per second for 30 seconds for CaGr, or every 10 seconds for 300 seconds for FITC-AV (due to the slower response of FITC-AV). A baseline PI image was acquired to identify a target cell as dead or dying. After five baseline images were acquired, TTL #2 triggered a Hewlett Packard (HP) 8112A pulse generator to deliver a series of TTL pulses to a custom transmission line pulser at 5Hz rep rate (max repetition rate of custom pulsing system). This custom pulser is capable of delivering electrical pulses with amplitudes from 1–999 V and with six discrete pulse widths: 10, 30, 60, 200, 400, or 600 ns. The pulses were then delivered to isolated cells positioned between a pair of 125 µm diameter tungsten electrodes separated by 100 µm. The electrodes were placed 50 µm above the glass surface to limit interference of the field profile. Sample traces of the pulse waveforms, collected by a Tektronix TDS3052 500-MHz oscilloscope, are presented in Fig. 1B.

The amplitude of the electric field was estimated using a three dimensional Finite Difference Time Domain (FDTD) model (Fig. 1C) [30], [31], as previously reported by Ibey *et al*. [26]. The model included a pair of two 125 µm diameter tungsten electrodes separated by 100 µm, angled at 30°, positioned 50-µm above the 180 µm thick glass coverslip and immersed in a 0.9% homogenous saline solution. A 2×2×2 µm voxel size was selected for FDTD modeling and dielectric properties of saline and glass were obtained from previous literature [32], [33]. The parallel electrodes were assumed to be perfect conductors and the steady state electric field amplitude at the glass surface was calculated using a trapezoidal pulse waveform from a voltage-gap source.

The generation of an electric field between two electrodes will generate a change in environmental temperature. To model the worst case temperature rise generated by repetitive nsPEF exposures, we assumed that the electrode system as a whole was approximately a 300 µm sphere of uniformly heated saline surrounded by an infinite homogenous saline solution. This approximation is assumed valid as both the height (4 mm) and width (35 mm) of the saline in the dish are much greater than the exposed saline volume. We solved for the steady state temperature after an infinite amount of 600 ns pulses at 3 MV/m being applied to the system at 5Hz repetition rate (the maximum parameters applied in our study) using Equation (1) [34], where ΔT is the steady state temperature rise (°C), E_D_ is the electric field amplitude (V/m), σ is the electrical conductivity (S·m^−1^), τ is the pulse width (sec), f is the repetition frequency (Hz), R is the radius of the heated spherical region (m), and k is the thermal conductivity (W·m^−1^·°C^−1^). The electrical conductivity of the exposure buffer with and without calcium was measured using a commercial conductivity meter (Mettler Toledo Sevenmulti, Switzerland) to be 1.381 and 1.354 S·m^−1 ^at 24°C, respectively. Using these values the maximum achievable temperature rise for an infinite number of pulse exposures at 5Hz is approximately 2.8°C.

### Confocal Fluorescent Imaging

The microscope was configured for 8-bit, DIC (Differential Interference Contrast) imaging using an EC Plan-Neofluar 40×/1.3 DIC Zeiss oil immersion objective with an optical section of 1 µm. The laser illumination levels and gain for the photomultipliers (PMTs) detecting the fluorophores were the same throughout. Cells which appeared to be in the process of division were avoided. All experiments were conducted at room temperature. Cells were exposed to the nsPEF setting at time, t = 0, and images of the FITC-AV were acquired simultaneously with the DIC image in 5-s intervals up to 3-min post exposure. Cell plates were never experimented upon for more than 1-hr in order to minimize significant environmental stress from the buffer, gas exchange, and/or prolonged room temperature exposure. When possible, cells were observed for changes in PS externalization at longer time intervals (t = 30 up to 60 min post nsPEF exposure). Cells were randomly exposed to a mixture of three discrete amplitudes (0.58, 1.13, and 2.27 MV/m), five discrete pulse widths (10, 30, 60, 200, 600 ns) and increasing pulse numbers (1–2000). Sham exposures were run to determine the rate of fluorescence change during imaging. Additionally, the change in fluorescence was measured in relation to the baseline fluorescence measurements per cell to account for any change in fluorescence due to the microscope and laser illumination over time.

To set the dynamic range for CaGr, a plate of cells loaded with CaGr were imaged with 10 µM ionomycin (Sigma Aldrich, I3909) (ionophore that increases intracellular calcium concentration) added to the outside solution (data not shown). We found that cells exposed to a single (n = 1) 600-ns pulse at a setting of 2.27 MV/m give a similar percent change as ionomyocin. Additional experiments were performed to determine the impact of changing exposure parameters, amplitude (A), pulse width (τ), and number of pulses (n),

### Probit Analysis

Acquired images were processed using MATLAB®. The 8-bit grayscale FITC-AV images were background subtracted, using the baseline images, then converted to binary images (threshold = 11). If a pixel identified to be on a targeted cell changed from a 0 to a 1 and persisted for more than 10 seconds, or two image frames (0.2 fps), then the cell was counted as a positive response (PS externalization occurred) to the nsPEF exposure settings (A, τ, n). If the binary images remained black, all 0 over time, then the cell was counted as a negative response (no PS externalization) to the nsPEF exposure. Thus cells were examined individually and classed as a 1 or 0 for a positive or negative response respectively. Binary data for a set of (A, τ) were then analyzed using Probit analysis to determine the ED50 for the number of pulses, n, needed to cause a positive read, within the 3-min observed time interval, in 50% of the population of cells.

Probit analysis is used for quantifying binomial data into a statistical response based upon the assumption the data fit a normal distribution [35], [36]. It is commonly used in toxicology and other areas of dosimetry where natural biological variability within a population is understood to exist. The analysis provides an estimate of the response threshold under investigation represented as a statistical chance (i.e., 50% likelihood) as a function of dose. Probit requires data to have responders and non-responders to the explored stimulus meaning that cells where no response was observed are as important to the outcome of the Probit results as the cells which did respond. On average, 52 individual cells were tested for each exposure (A, τ) pairing on the pulser’s settings in order to obtain reasonable 95% confidence intervals (CI) for the Probit results.

## Results and Discussion

**Figure 2:**
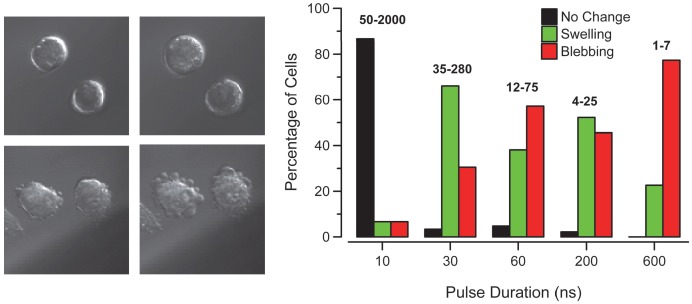
Example images of cellular swelling and blebbing are shown to depict the categorization used in the visual analysis. The graph (right) shows the observed changes in cellular morphology with increasing pulse width and decreasing number of exposures at a set exposure amplitude of 2.3 MV/m. It appears that fewer 600 ns pulses preferentially cause cellular blebbing, while shorter pulse widths favor swelling. Almost no change in morphology was observed in response to pulses with 10 ns duration.

Changes in cellular morphology were observed in the DIC images with increasing pulse number and shortened pulse duration. To simplify the analysis of the morphological response to nsPEF, we chose to categorize observations as no change, swelling, or blebbing using DIC images of each exposure. Fig. 2 shows the general trend apparent in cells exposed to high pulse number, short duration (10 ns) versus low pulse number long duration (600 ns). We present two images, before and after nsPEF exposure, of a cell “swelling” (top) and blebbing (bottom). The graph shows the percent of the total exposed cells in each of the three categories. The exposure amplitude was 2.3 MV/m for all exposures. We observed that at 10 ns with up to 2000 pulses, very few cells showed any changes in cellular morphology. At 30, 60 and 200 ns exposures, we observed morphological changes in almost every exposed cell. The trend favors swelling at lower pulse durations and blebbing at high durations. At 600 ns, the trend favors blebbing at even a single pulse exposure. We observed that cellular swelling is favored for fewer pulses or shorter pulse durations, whereas blebbing appears to dominate at longer pulse-durations or more pulses. We believe that such a trend would hold true for 10 ns, but due to the limits of our exposure system, we are unable to surpass 2.3 MV/m per single pulse. Other research groups have shown cellular responses to exposures as short as 4 ns with field intensities nearing 8.0 MV/m [37]. Furthermore, blebbing may be favored at high enough amplitudes even for very short duration pulses. These observations suggest the morphological response (swelling vs. blebbing) to nsPEF may be related to pulse width with successive exposures acting to exacerbate the effect.

**Figure 3:**
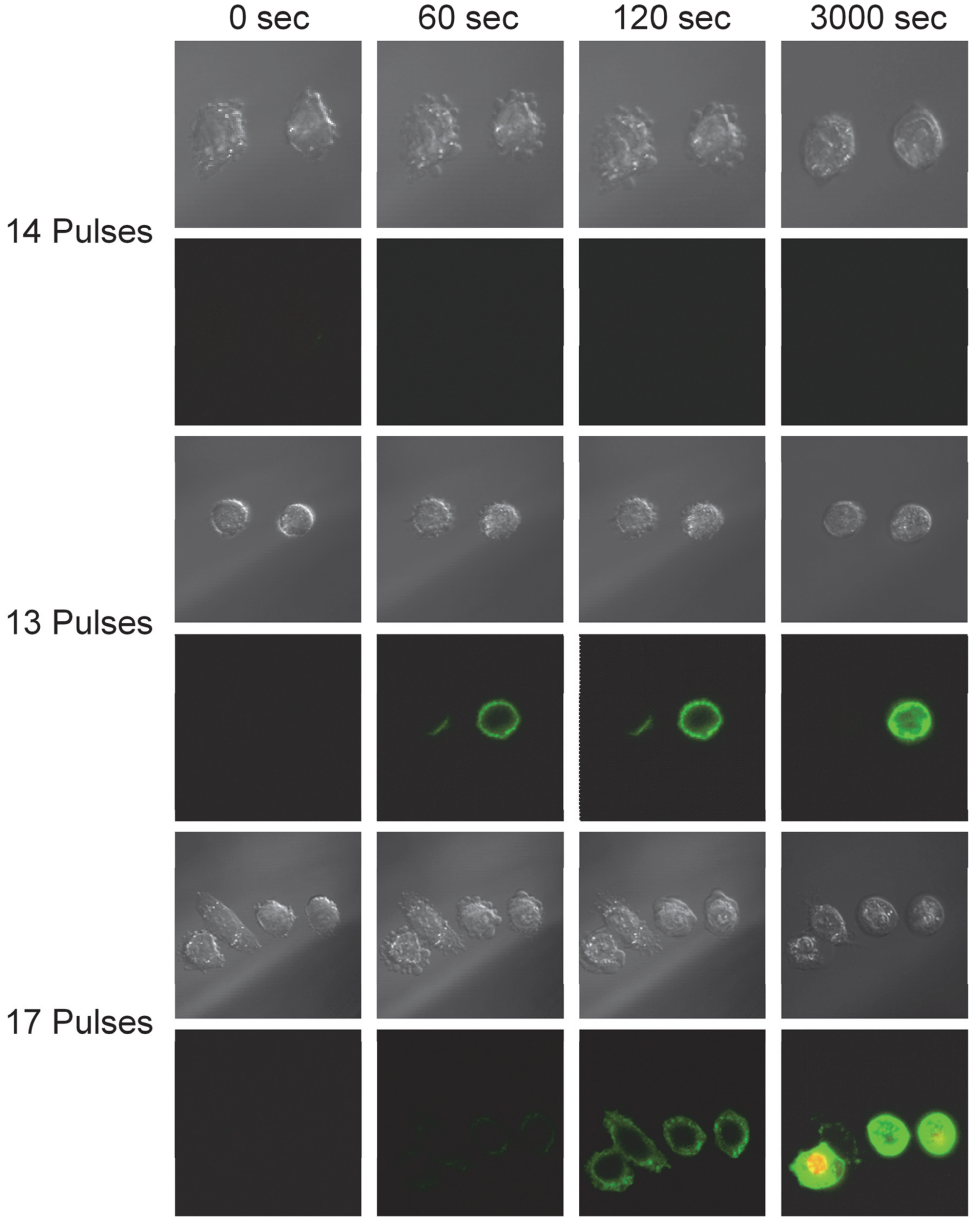
Representative images of the diverse reaction of CHO-K1 cells to nsPEF exposure. Series 1: Cells undergoing morphological changes without the observation of PS externalization. Series 2: Two cells both expressing PS acutely, but one cell recovers while an adjacent cells remains expressive at 50 minutes post exposure. Series 3: Four cells exposed to the same conditions, all indicate PS expression, but only one cell expresses PS initially and overtime uptakes PI into the interior of the cell.

The degree of FITC-AV expression (PS externalization) to different nsPEF parameters is highly variable. Fig. 3 shows examples of three exposure paradigms with dramatically different cellular responses. The first series in Fig. 3 shows two cells exposed to fourteen, 600 ns pulses at 1.13 MV/m. At 60 seconds post-exposure, blebs have formed which persist after 120 seconds. Images of the same cells at 3000 seconds (50 minutes) show a round morphology, but continue to lack observable FITC-AV expression or PI uptake. The second image series in Fig. 3 presents two cells exposed to thirteen, 600 ns pulses at 1.13 MV/m with a different response. Within 60 seconds post exposure, we observed cathodic expression on the left cell and whole cell expression on the right cell. At 120 seconds, expression appears to have increased. However, at 3000 seconds, we observed full recovery in the left cell and persistent expression in the right cell without PI uptake. Pore formation from nsPEF has been known to be asymmetrical with PS externalization occurring largely on the anodic side of the cell, but for much shorter pulse durations (<100 ns). [38]. This asymmetrical response could be due to variations in lipid composition [39], resting potential [40]and possibly the permanent dipoles of the lipids [41]. Asymmetry in nsPEF response has also been demonstrated to be dependent on the cell type [27], [42], [43], though we used the same cell type throughout this study.

In the last image series in Fig. 3, four cells were exposed to seventeen, 600 ns pulses at 1.13 MV/m. In this experiment we again observed anodic expression after 60 seconds post exposure, which by 120 seconds appears observable at both the anodic and cathodic poles of the cell. At 300 seconds, we observed full cell expression followed by large PI uptake by 3000 seconds. The series imaged in Fig. 3 show a general dose trend for PS externalization, but also emphasize the dichotomy of expression even amongst cells which experience a similar dose in both acute response and long-term recovery. For the purposes of this study, we only evaluated cells for FITC-AV expression after one and no more than 300 seconds post exposure to eliminate delayed effects. That being said, cells with acute FITC-AV expression occasionally showed very abrupt (within 15 seconds) and intense whole cell FITC-AV expression beyond 5 minutes. We suspect this occurrence is related to an active (energy limited) response to cellular swelling, presumably initiated by the formation of pores in the plasma membrane [4]. We hypothesize that when the cell can no longer actively respond (out of existing energy stores) to the ion imbalance, it swells and the entire plasma membrane abruptly loses asymmetry. Experiments focused on tracking ATP depletion are planned to validate this hypothesis.

**Figure 4:**
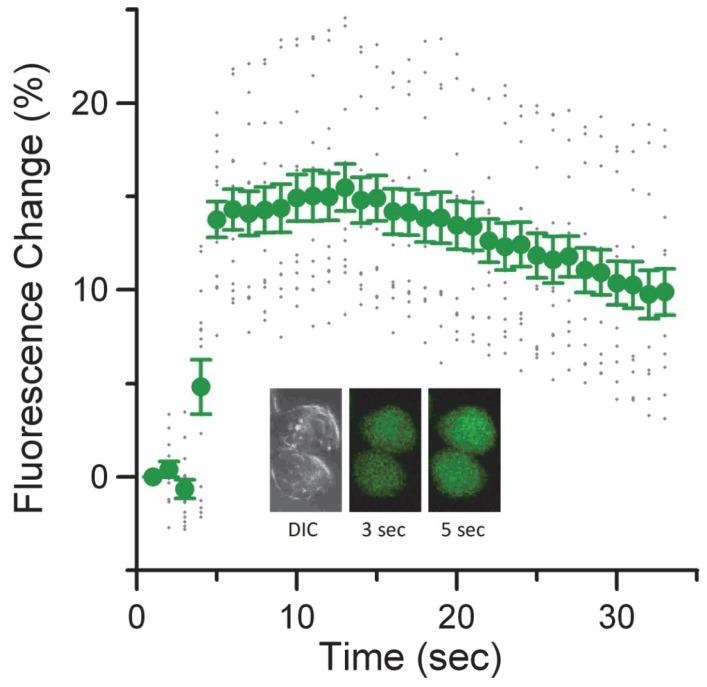
Rapid and sustained calcium influx following nsPEF exposure. Graph depicts the average response of ten CHO-K1 cells upon exposure to a single 600 ns electric pulse at 1.8 MV/m applied field. Inset shows a representative response from two cells just before (3sec) and immediately after the pulse (5 sec). Errors bars represent the standard error of the mean.

To elucidate the dose response for calcium uptake in cells exposed to nsPEF, we preloaded cells with calcium green indicator (CaGr). Figure 4 shows the rapid and sustained response in the whole cell fluorescence that indicates a change in intracellular calcium following a single 600 ns, 1.8 MV/m pulse exposure. The FDTD-predicted electric field was recalculated because the space between the tungsten electrodes was slightly wider for CaGr experiments, resulting in a predicted electric field of 1.8 MV/m as opposed to 2.3 MV/m. It is important to note that the calcium rise occurs rapidly (under a second) and the intracellular fluorescence remains elevated for at least 30 seconds after the response (the final image we acquired). This influx is quite rapid when compared to the slow response of Annexin V, which takes tens of second to be detectable [11]. It remains unclear whether the slower response of FITC-Annexin V is due to binding kinetics of the dye or slow externalization of PS residues. However, for intense exposures, we observe some externalization within seconds of exposure that builds up slowly over time.

**Figure 5:**
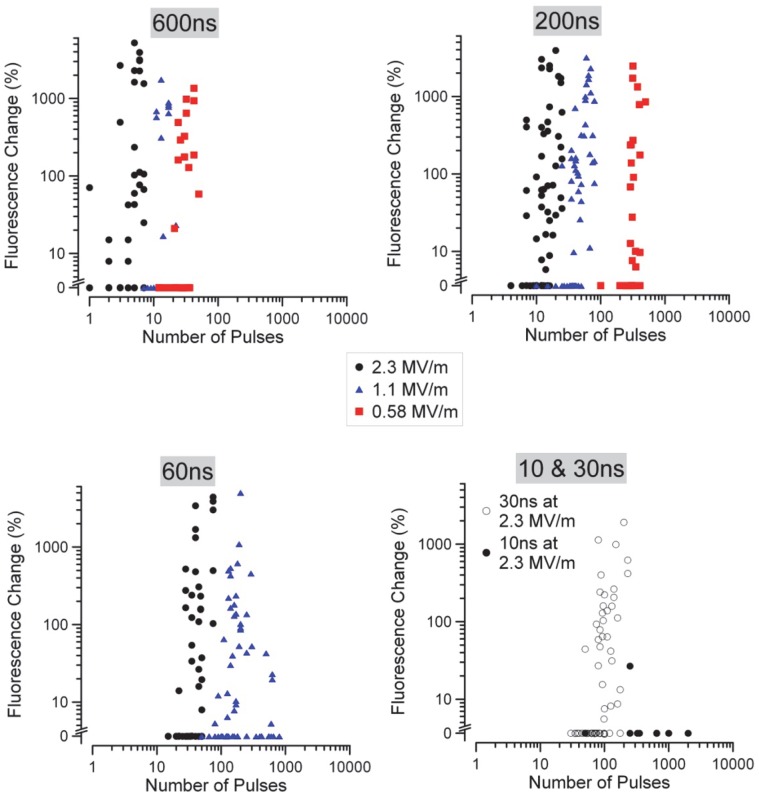
Graphical representation of the raw FITC-AV fluorescent change in cells exposure to multiple nsPEF at three amplitudes. Data are separated by pulse width for clarity. A trend of higher amplitude exposures requiring fewer pulses to cause externalization of PS is evident at all pulse widths. However, the dichotomy of the response is also evident as demonstrated by the large spread in the data at each amplitude.

As shown in Fig. 3, the cellular response to nsPEF-induced FITC-AV expression is highly diverse. This diversity may be due a variety of cellular and environmental factors, including proximity to neighbors, stage of mitosis, or subtle morphological differences. Fig. 5 shows the change in FITC-AV expression upon exposure to nsPEF at five discrete pulse widths (10, 30, 60, 200, 600 ns) and three amplitudes (2.3, 1.15, and 0.575 MV/m) versus the number of applied pulses. From these data, a dosimetric trend appears despite the diversity of the response (shown by the data spread at each amplitude). At lower pulse widths, 60, 30, and 10 ns, 0.58 MV/m amplitude pulses were applied, but positive responses could not be obtained. In general, the data suggest that increased FITC-AV expression is achieved by more pulses and higher voltage, which agrees well with previous observations [10].

**Figure 6:**
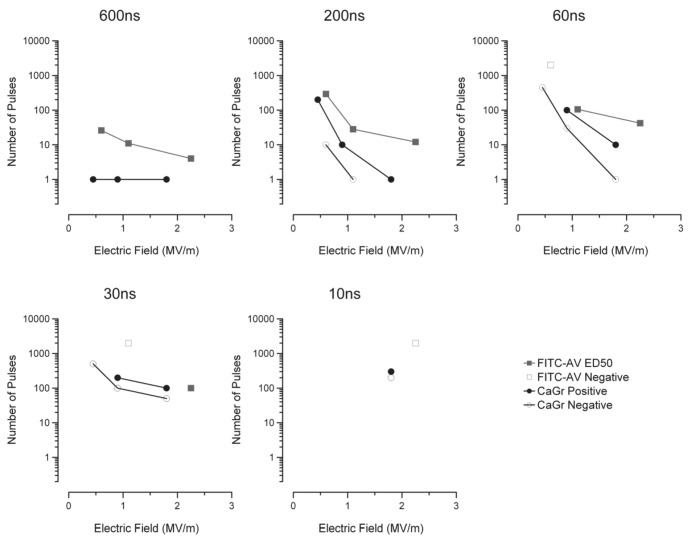
Probit predictions of the ED50 (squares) for PS externalization based on binary categorization of the raw data presented in Fig. **5.** Additionally, the data points representing the observed level of calcium uptake by the cells using CaGR are presented (circles). In cases, the number of pulses for the ED50 of PS externalization (FITC-AV) was above that required to observe calcium uptake (CaGr). Positive PS externalization was not achieved for 10 ns exposures and 30 ns at 1.1 MV/m.

By applying Probit analysis to the data presented in Fig. 5, the ED50 (expected number of pulses to cause 50% of the cells to yield a FITC-AV expression) can be predicted for a given amplitude and pulse width. The number of pulses versus the electric field (MV/m) for each pulse width is presented in Fig. 6. As expected from the raw data, the number of pulses required to achieve PS externalization (gray box, ED50) increases with decreasing pulse width. Empty squares represent exposures that were attempted but did not elicit acute PS externalization. As both the pulse width and amplitude decreased, the number of pulses required to achieve a positive response grew considerably All exposures were delivered at 5Hz, so no attempt was made to go beyond 2000 pulses due to the time required.

Using CaGr to indicate calcium uptake in exposed cells (black circles in Fig. 6), we repeated our experiments and found that for 600 ns, all amplitudes were sufficient to cause uptake of calcium with one pulse. The electric field amplitude for the CaGr experiments, modeled by FDTD, was slightly lower for all exposures due to a change in the distance between the electrodes. For 200 and 60 ns exposures, more than a single pulse was required to cause uptake of calcium (Fig. 6). Exposures that did not result in uptake of calcium, as indicated by CaGr, are shown as gray circles in Fig. 6. PS externalization proved difficult to achieve for the 30 and 10 ns pulse durations, while uptake of calcium was readily observed. In all experiments with the CaGr dye, the uptake of calcium occurred below the ED50 for FITC-AV expression, suggesting calcium moves into the cell even though PS may not externalize.

We were concerned that the high concentration of FITC-AV required for rapid live cell analysis of PS externalization may alter the impact of nsPEF on the cell, as compared to CaGr experiments where no FITC-AV was present. Thus, we conducted a series of exposures in which cells were loaded with CaGr and doped with Alexa 680-AV (Alexa 680 was chosen to avoid spectral overlap with CaGr) and PI at concentrations consistent with our other experiments. By tracking both PS externalization and CaGr, we found there was no change in the number of pulses required to generate a positive response from each dye (data not shown). This experiment confirmed that the endpoints observed through separate experiments were not biased by the high concentration of FITC-AV.

**Figure 7:**
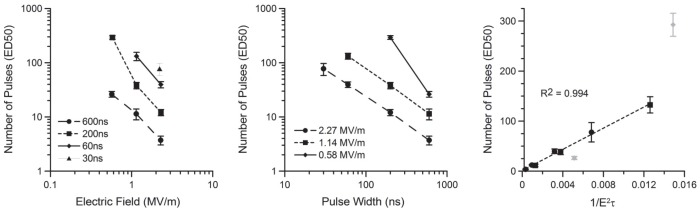
Graphical representation of ED50 PS externalization thresholds for all amplitudes (left) and pulse widths (middle). Using the inverse of the square of the electric field multiplied by the duration of the exposure, we arrive at a model to predict the externalization of PS on the outside of the membrane across a wide parameter space. Exposure amplitudes corresponding to 0.58 MV/m did not fit this model well despite maintaining the general trend.

**Figure 8:**
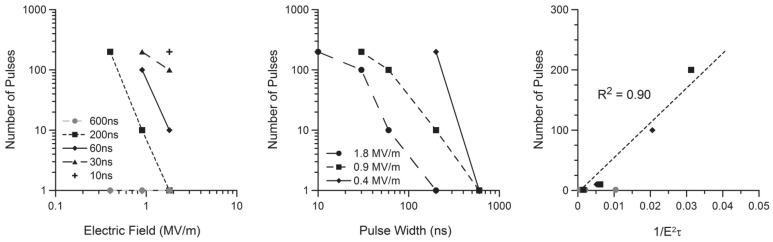
Graphical representation of observed calcium influx for all amplitudes (left) and pulse widths (middle). Using the inverse of the square of the electric field multiplied by the duration of the exposure, we arrive at a model to predict the calcium uptake threshold over a wide parameter space (right). All 600 ns exposures were ignored in generating the model as all electric field amplitudes produced an observed calcium influx.

A trend in the ED50 FITC-AV data can be determined (Fig. 7) for pulse amplitude (left) and pulse width (middle). In both graphs, a parallel response can be seen at 1.14 and 2.27 MV/m, suggesting a mathematical correlation between these data. By excluding the lowest amplitude exposures, which lacked many positive responses, we found a high correlation between the number of pulses and the inverse of the square of the electric field (E_D_) and pulse width (τ). This relation matches very well with previous work using patch clamp electrophysiology, which showed that the permeability of the plasma membrane upon single pulse exposure depended on the absorbed dose (AD = E_D_^2^·τ·n) [28]. A second paper focused on the impact of multiple pulses on membrane permeability and described a “diminishing return” with successive exposure [44]. However, in this current experiment, significantly higher amplitudes were used to obtain FITC-AV expression as compared to those tested in previous papers. When we applied the same analysis to the calcium response data, we observe a similar trend (Fig. 8). However, to generate this trend, we excluded 600 ns exposures as even a single pulse at 0.4 MV/m causes measureable calcium influx. This result reinforces the observation that calcium influx occurs at lower doses than externalization of PS.

Pore formation in, and molecular uptake of macromolecules through, the plasma membrane have been reported to be non-linear [29], [45]. Vernier *et al*. [21] examined the effects of PS externalization from nsPEF at various pulse repetition rates and found that repetition rate does affect observed PS externalization. Vernier *et al*. suggested the idea of superposition to describe whether the plasma membrane may or may not return to initial state in between pulses depending on the exposure parameters frequency (f), n, τ and E_D_, which influence pore lifetime. Vernier *et al*. hypothesized that a threshold membrane voltage may need to be achieved in order to initiate PS externalization, a threshold which could be unachievable at smaller n, τ, E_D_ or f. This hypothesis may explain why we did not observe PS externalization in any cell exposed to 2000 pulses of 2.27 MV/m, 10 ns; 1.14 MV/m, 30 ns; or 0.58 MV/m, 60 ns. It is possible that PS externalization may be observed at these field settings if we increased the pulse repetition rate; however, we held the repetition rate constant at 5Hz, the maximum rate for our pulsing system.

**Figure 9:**
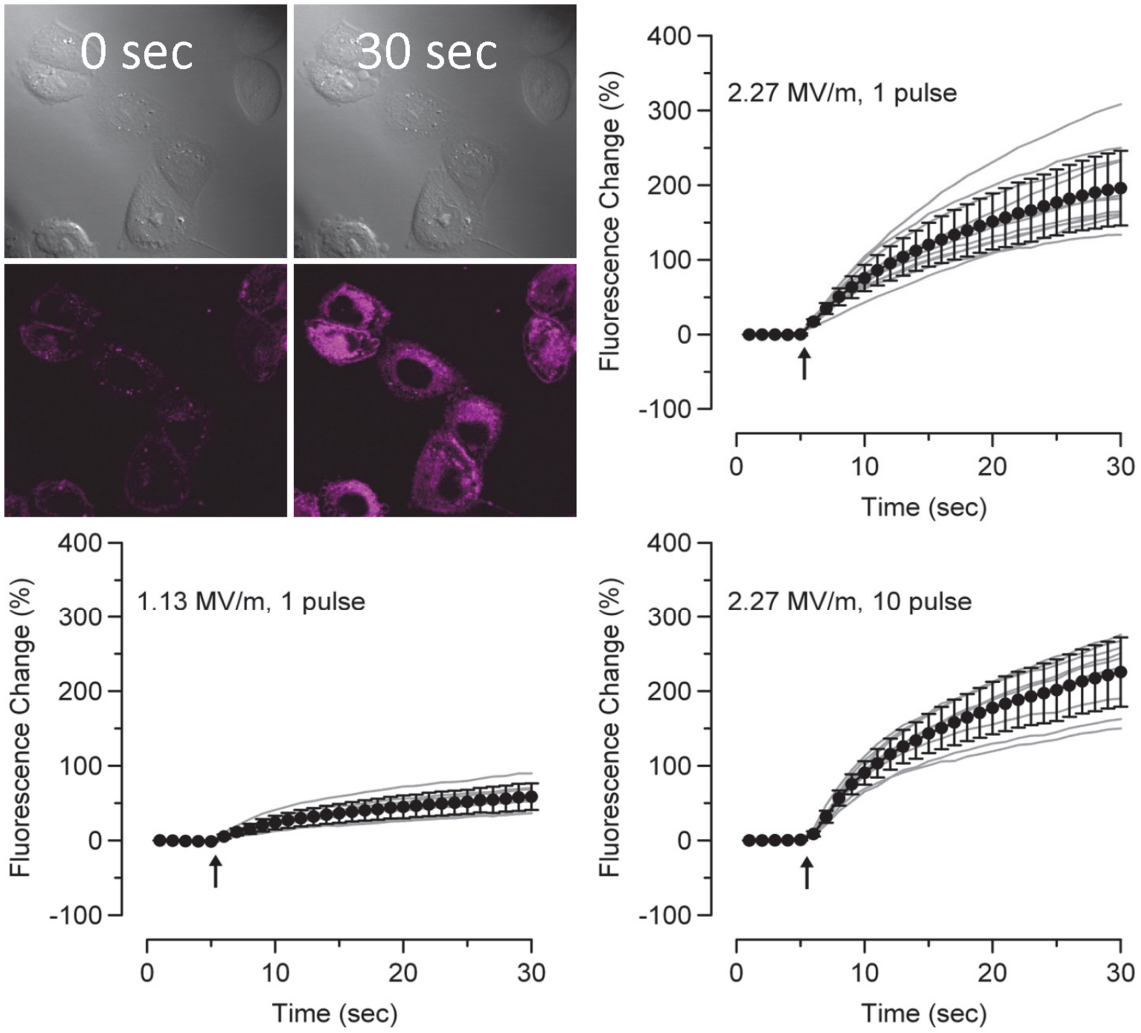
FM1-43 fluorescence change upon nsPEF exposure. A representative image of FM1-43 uptake in cells exposed to 1, 600 ns pulse illustrated by considerable dye entering the interior of the cell observed under confocal microscopy. Traces of the whole cell fluorescence during nsPEF exposure show that one and ten pulses at 2.27 MV/m cause immediate uptake of FM dye into the cell. Single pulse exposures at 1.13 MV/m show a reduced but observable increase in FM1-43 fluorescence.

If PS externalization occurs by lateral diffusion through pores, then it would stand to reason that smaller pore diameters may energetically limit this translocation. Therefore, induced PS-externalization is more likely to occur for pores of larger diameter supporting a possible relationship between intensity of exposure and degree of externalization. In this paper, we found that such a relationship does exist, but falls apart at lower amplitude exposures, those closer to previous whole cell patch work. This transition point could represent a minimum pore diameter necessary for lateral diffusion of membrane phospholipids. To further support this hypothesis, we used FM1-43, a surrogate dye for Annexin V previously used to detect externalization of PS, albeit non-specifically [25]. Contrary to previously published results for much shorter pulse widths (<20 ns), 600 ns single pulse exposures not only caused a nearly instant increase in FM1-43 fluorescence at the membrane facing the electrodes, but an immediate whole cell uptake was observed. Fig. 9 shows CHO-K1 cells before and after exposure to a single 600 ns pulse. The mean whole-cell fluorescence after exposure to one 600 ns pulse at 2.27 and 1.13 MV/m and ten, 600 ns pulses at 2.27 MV/m are presented. FM1-43 expression is immediately seen in all exposures, with one and ten pulses showing a similar increase. Single exposures at half the exposure amplitude caused a smaller, but still significant, response. These data suggest that single pulses at 1.13 MV/m may cause the formation of pores within the membrane that are the diameter of the FM1-43 molecule (1.41 nm) [46]. High amplitude single pulse exposures cause maximal uptake of FM1-43 dye, suggesting that many pores of sufficient diameter were likely produced. However, it remains unclear how FM1-43 enters the cell with possibilities including direct diffusion through the lipid membrane, endocytosis, or through a pore or ion channel. We postulate that FM1-43 is entering the cell via a pore as the response is rapid and field dependent. Additional pulse exposures did not further increase the cellular response. When compared to the externalization of PS, we observed a large fluorescent response by a single 600 ns pulse, whereas three to four pulses were required to induce PS externalization. These observations show that PS externalization and FM1-43 dye response are not detecting similar fundamental changes in the plasma membrane.

### Conclusions

In this paper, we explored the dose response relationship between nsPEF exposure parameters and expression of FITC-AV on the plasma membrane surface, demarking the externalization of PS residues from the inner membrane leaflet. We have shown that increasing the pulse duration, number of pulses, and pulse amplitude increases the probability of PS externalization. We also found that persistent extracellular uptake of calcium occurred with fewer pulses and lower amplitude exposures than PS externalization. Finally, we monitored the increase in FM1-43 fluorescence and found that single exposures were capable of causing whole cell uptake of FM1-43, whereas multiple exposures were required to express FITC-AV on the membrane surface. By capturing this dose response data, we believe that the uptake of calcium into the cell is not directly related to the induced externalization of PS. This suggests that calcium-dependent activation of scramblase is unlikely solely responsible for nsPEF-induced PS externalization. At low intensities, we do observe loss of FITC-Annexin V fluorescence, indicating membrane recovery many minutes after exposure. This result suggests that ATP-dependent enzymes responsible for maintaining membrane asymmetry remain functional (flippase/floppase). Additionally, by showing the persistent uptake of both calcium and FM1-43, theorized to be through pores created during the nsPEF exposure, we show that pores with diameters as large as 1.5 nm (diameter of FM1-43) may be present without observable PS externalization. This finding suggests that if PS externalization occurs by lateral diffusion of phospholipids, a minimum separation between adjacent phospholipids is required to overcome the molecular forces that otherwise prevent diffusion through smaller pores. This requirement may point to a direct correlation between pulse length and induced pore diameter and allow control of the membrane breakdown by tuning the pulse parameters. Based on these observations, future efforts should focus on determining the relationship between pore diameter and PS externalization.
